# Category bias and front-of-package labelling

**DOI:** 10.3389/fpubh.2026.1963968

**Published:** 2026-10-27

**Authors:** Iina Ikonen, Florian Buehler

**Affiliations:** 1 Department of Marketing, Faculty of Economics and Business, University of Groningen, Groningen, Netherlands; 2 Vorarlberg University of Applied Sciences, Dornbirn, Austria

**Keywords:** food policy, front-of-package label, healthy food, nutrition label, public health

## Abstract

**Background:**

Front-of-package nutrition labels are commonly used to offer consumers more information about a food product’s healthfulness. However, not much is known about how broader category perceptions influence consumers’ interpretation of objective nutrition information or how individual characteristics might influence this.

**Methods:**

We report the results of five experiments in which participants were presented with the same nutrition label, but told it was associated with a product from either a more or less healthy product category. They rated the product on its overall healthiness and the level of specific nutrients.

**Results:**

Across the five studies, we find that consumers evaluate the same nutrition information very differently across different categories. Even though they are able to adapt their evaluations based on the nutrient levels presented, the category effect remains. We further find initial evidence that the category bias is stronger among more health-conscious consumers. However, subjective or objective nutrition knowledge or one’s confidence in their nutrition knowledge do not help overcome the bias.

**Conclusion:**

All in all, the results suggest that nutrition information is interpreted in the broader context of food categories. Policy makers and consumers themselves need to find ways to increase their focus on individual products over broad categorizations.

## 1 Introduction

Front-of-package nutrition labels have become prevalent in the marketplace, building on the idea that offering consumers simpler and more accessible information about the healthiness of a product will enable them to make better choices. Most such labels are based on providing objective details about the nutritional value in a summary format on the front of the food package, for example by presenting the per-portion levels of calories, sugar, saturated fat and sodium in a simplified icon (1). Often, providing consumers with more information about a product’s healthiness is seen as the key to improving consumers’ nutritional knowledge and promoting healthier products. However, judging a product’s healthiness based on such information is not straightforward as consumers still need to *interpret* this objective information to draw inferences about the presented levels being relatively healthy or unhealthy.

This interpretation does not happen in a vacuum: consumers interpret the objective information presented to them in relation to their existing knowledge and expectations towards the product, for instance based on the product category (2). The question in consumers’ minds might not be “Are 100 calories a lot?” but “Are 100 calories a lot for this type of product?” For example, consumers generally believe that buying a salad is a healthy choice given the healthiness of the category, and may not realize that an option with cheese and a heavy dressing might in fact be rather unhealthy (3). Based on this, we argue that the *same* objective nutrition information might lead to different interpretations depending on the product it is placed on. Thus, stating “200 calories” on a label may be perceived as low when placed on a chocolate bar but as high when placed on a fitness granola bar.

Food type has been shown to have a strong influence on healthiness perceptions. Consumers easily categorize foods into groups of “healthy” and “unhealthy” or vices and virtues (4, 5). Food category or type has been found to be a primary attribute in influencing consumer perceptions about the food because a categorization is easier to process than a continuous facet (e.g., level of calories; 3). In fact, when a product is considered healthy in one aspect, this positive perception often is overextended to other factors resulting in more positive evaluations of the product overall, creating a so-called health halo (4). Thus, if a product category overall is perceived healthy, also specific attributes of individual products within this category might be perceived in line with this category expectation. In fact, even a perception of a brand as healthy can lead to mis-estimations of calories (6). However, all in all, more research to understand such effects is needed as “little research has addressed how perceptions of a superordinate attribute (i.e., a “healthy” product) may differentially bias inferences of specific subordinate food attributes (calories and nutrients)” (6). Our research extends this line of work by explicitly examining how such category expectations influence consumer interpretation of FOP labels, even when objective nutrition information is present.

As a possible solution, it has been suggested that objective nutrition information can reduce such health halos and help overcome some of these biases (7, 8). However, this has been specifically tested in comparative settings, where consumers were directly able to compare alternatives and see which product was healthiest. In reality, this finding may have less impact on real consumer choices, as consumers face severe time constraints when shopping for groceries and very few consumers have time to compare multiple products on their nutrition values (7, 8). For instance, when standing in front of the cereal aisle in a supermarket, most shoppers will not pick up three different boxes to compare in detail calories per serving, but rather glance at one option and make a quick decision. This may be the case even more so in online settings, where finding the nutrition information requires clicking through to the product page, scrolling to the bottom of it for the nutrition information, and then going back to the category page to find a different product for comparison. Situational factors and a general shift in shopping behavior to more online purchases may thus decrease consumers’ ability to compare products directly even more (9). Therefore, it is crucial to understand how consumers interpret identical nutrition information in single-product exposures, which represent the most common real-world shopping situation. This highlights the need to study not only whether labels are noticed, but also how they are interpreted once they are.

This becomes even more crucial knowing that manufacturers can change categorizations, and thus the expectations of healthiness, of their products often by changing the naming or description of their product. For example, the same product might be called energy *water* or energy *drink* – does this influence how consumers interpret the nutrition information? When Finland trialled a product category-based sugar tax, manufacturers increasingly brought to market cookies (which were not taxed) rather than chocolates (taxed; 10), often in the same flavors and with only small changes in the product, showing the ease with which companies may influence the categorization of their products. Such practices illustrate that category-based interpretations are not only theoretically interesting but also highly consequential for policy and public health. If the product category influences the interpretation of objective nutrition information as we expect, there are strong implications to the effectiveness of front-of-package labelling initiatives. All in all, we propose that consumers interpret identical FOP nutrition information differently depending on the product category it is associated with, a phenomenon we call category bias in nutrition label interpretation. This extends prior work on health halos by showing that even when objective nutrition information is present, category expectations continue to shape judgments. We further examine whether this bias is moderated by consumers’ health consciousness and nutrition confidence. Across five experiments, we test these hypotheses in varied product contexts.

## 2 Theoretical background

As traditional nutrition information panels (or nutrition facts panels), typically found on the back or sides of food packaging, have been found insufficient in guiding consumers towards healthier choices in the supermarket, front-of-package (FOP) nutrition labels have been developed and growing in popularity (11). The idea behind these FOP labels is to offer simpler information that is easy and fast for consumers to understand (12). However, even though such labels have attracted a lot of attention from researchers in numerous academic fields (see, e.g., 1, 13, 14 for reviews), it remains unclear whether these labels are, in fact, effective in driving healthier choice. This discrepancy highlights the need to study not only whether labels are noticed, but also how they are interpreted once they are.

One reason for the mixed findings of previous research may relate to the way personal or situational differences influence consumers’ attention to and reliance on nutrition labels. For instance, van Herpen and van Trijp (15) found that consumers are less likely to be pay attention to objective nutrition information when making decisions under time constraints. This would imply that factors such as stress, motivation and type of shopping trip may have significant impact on FOP label effectiveness, though such factors are often not accounted for in previous studies. In this research, we propose that because consumers typically are under some level of time constraint or are dealing with information overload when shopping, their interpretation of nutrition information is based on their pre-existing knowledge.

It has been well established that consumers somewhat automatically categorize products into simpler groups (16). In the context of food, different options are often considered to be relatively healthy or relatively unhealthy, in other words virtues or vices (17, 18). As products are often evaluated as examples of the broader category they belong to (2), expectations regarding a product’s level of healthiness are likely to be related to perceptions about the overall category. In fact, consumers do hold strong expectations about products’ nutrition values, which can influence post-purchase evaluations, calorie estimates and even review behavior (2, 19–21). These category-based expectations can therefore act as cognitive schemas, guiding how subsequent data such as FOP nutrition information is processed and evaluated.

As mentioned, FOP nutrition labels, despite being in simpler formats than nutrition facts panels, still typically require the consumer to interpret the given information to some extent. While the FOP label makes it quick and easy to see that a product has, for example, 100 kilocalories per portion, it depends on a number of factors whether or not this is a desirable (relatively healthy) level or not. When making fast decisions, such as in a shopping environment overloaded with options and decisions, consumers rely on quick heuristics and mental shortcuts (22). Because the categorization of foods into healthy and less healthy is a common and simple process for most consumers, it is likely that the pre-existing knowledge about the product category will influence the consumer’s interpretation of the nutrition information (23). In other words, consumers may not read FOP numbers in isolation but through the lens of their category-based expectations.

Confirmation bias theory (24) suggests that consumers are likely to search out information that supports their pre-existing assumptions or interpret it in a way that does not require changes in the existing knowledge. In the food domain, consumers have been found to, for example, selectively focus their attention on information regarding their main decision-making criterion (e.g., healthiness or tastiness), and consider arguments on alternative factors as less convincing (25). For instance, consumers’ perceptions of plant-based meat alternatives as healthier than regular meat have been found to persist regardless of nutrition information being present (26). The authors found that promoting active comparison of nutrition values can help mitigate such a bias, but as discussed above, the time constraints and stress consumers face during a shopping trip and differing formats of nutrition information disclosure make such comparisons highly effortful and can result in a high reliance on intuition. In such cases, category-based expectations provide an accessible shortcut, making it likely that nutrition labels are interpreted through the lens of whether the product is seen as generally “healthy” or “unhealthy.” Thus, our theorizing connects health halo research with confirmation bias theory: even in the presence of objective information, interpretation remains biased by prior categorical beliefs.

Because of this, we propose that consumers will interpret the information on a FOP nutrition label such that products from categories generally considered healthy by consumers are evaluated healthier than those from less healthy categories even when the objective nutrition information is *equal*. More formally,

*H1*. Consumers will evaluate the same nutrition information as healthier when it is associated with a product from a healthy (vs. less healthy) category.

### 2.1 Strength of prior expectations

It is likely that consumers differ in the strength of their knowledge or expectations regarding specific food categories. Some consumers hold views regarding a food product’s healthiness that are more persistent in nature, while others may be more flexible in adjusting their views based on new information from different sources. Holding strong beliefs or overconfidence has been found to correlate with one’s tendency to exhibit confirmation bias (27). While more thorough processing has been argued to reduce such biases (28), others suggests that analytical processing can at times reinforce prior beliefs by providing more elaborate justifications for them (29). This suggests that the effect of strong beliefs on bias may be amplified based on whether new information is perceived as consistent or threatening to existing beliefs.

Food and nutrition is an area where many consumers indeed hold strong beliefs because it is a context that is personally relevant and relatable to all people. Food consumption and health are topics that consumers come across many times each day, and people hold specific, but often also diverging views about which factors impact a food product’s healthiness (30). Such firmly held views are known to foster confirmation bias, as consumers are more likely to interpret new information in ways that align with their existing beliefs rather than revise them (28, 31). Accordingly, consumers who see themselves as particularly health conscious or confident in their nutrition knowledge may be especially prone to filtering FOP information through their existing schemas.

We propose that consumers who are more persistent in their views about nutrition and healthiness of food products will also express the category bias of nutrition labels more strongly. In particular, consumers who perceive themselves to be highly health conscious and are very confident in their own nutrition knowledge are likely to feel that their own initial judgment of a product is sufficient to judge an item’s level of healthiness, rather than rely on details provided in the nutrition label. We therefore hypothesize the following:

*H2*. Consumers with stronger nutrition beliefs (e.g., higher subjective health consciousness or nutrition-related self-confidence) will express the category bias described in H1 more strongly than those with weaker nutrition beliefs.

We test these predictions using different products and settings across five experimental studies. Studies 1 and 2 establish the main effect that when seeing the same nutrition information, consumers evaluate a product as being healthier overall as well as in terms of the levels of specific nutrients when the label is associated with a product from a healthier (vs. less healthy) category. We also show that the effect is not due to a lack of understanding the nutrition label (Study 2) and that the effect replicates even within a category when perceptions of that category are primed to be more or less healthy prior to exposure to the nutrition label (Study 3). We find initial evidence for H2 showing that the effect is stronger among those who report higher subjective health consciousness in Studies 1 and 2. However, when using nutrition quizzes in Studies 4A and 4B, we do not find that this is related to either subjective or objective nutrition knowledge or the level of (over)confidence in one’s nutrition knowledge. We discuss the importance of our findings and call for more research into the role of health consciousness in the General Discussion section. All data and materials are available on ResearchBox^1^.

## 3 Study 1

The purpose of Study 1 was to establish the main effect that consumers will perceive the same nutrition information more positively when it is said to be associated with food category typically considered more (versus less) healthy.

### 3.1 Study design

Participants (250 undergraduate students, M_age_ = 19.28, 32.8% female) took part in a single-factor two cell between-subjects experimental design (product category: potato chips versus granola) as part of a research session containing multiple unrelated studies. The sample size in Studies 1–3 was determined by the availability of participants in the subject pool of the behavioral lab. We selected these two categories based on previous research contrasting healthy and less healthy foods (e.g., 32, 33). While the categories are generally perceived as clearly healthy versus less healthy, it is possible to find products with similar nutritional profiles, even if such products may not be representative of the category average. In follow-up studies we select products where similar nutritional profiles are more likely to be common.

In the experiment, participants were asked to imagine being in a supermarket looking for something to eat between meals and coming across either a bag of potato chips or granola. They were told they noticed some nutrition information on the package and were then shown a Facts Up Front style front-of-package nutrition label. The label stated that the product contained 140 calories, 2 grams of saturated fat, 2 g of salt and 4 g of sugar per 30-gram serving, in line with the four mandatory nutrients to include when using the Facts Up Front label (Figure 1). The label was identical across conditions. Participants were not shown the whole packaging of the product but just told that the label was on a product from their assigned food category.

**Figure 1:**
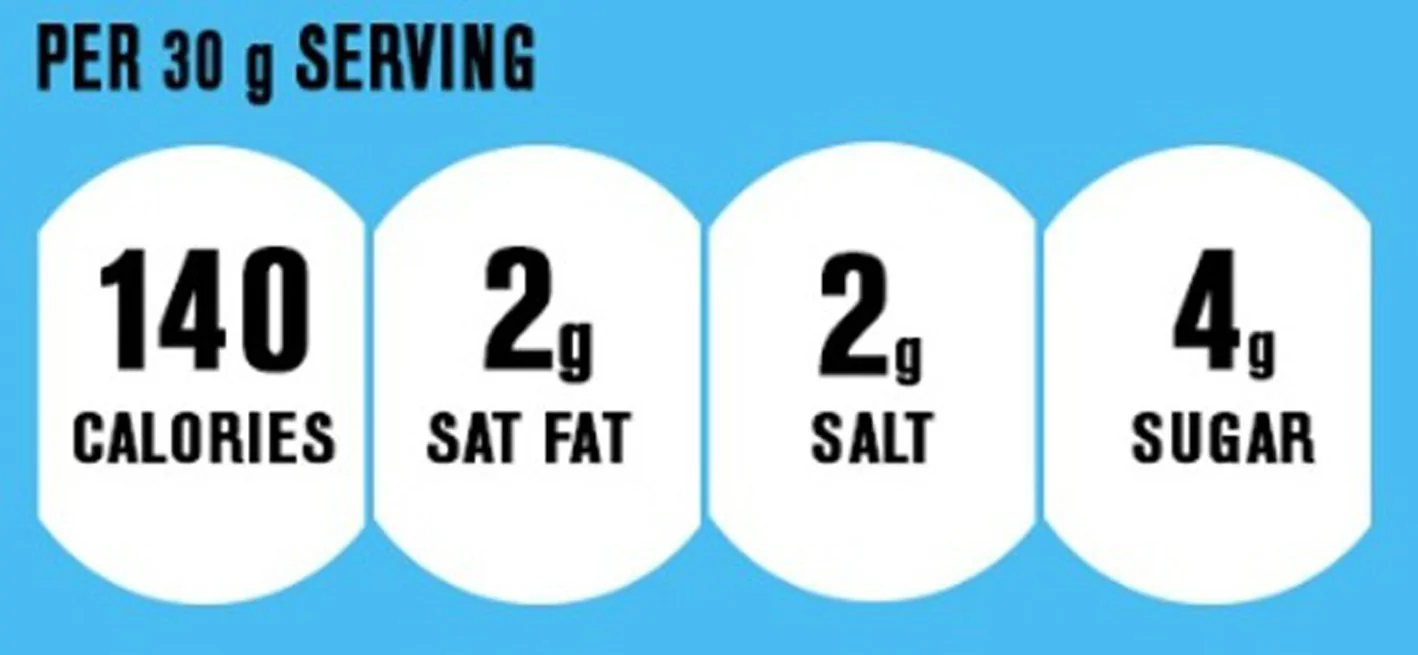
Front-of-pack label shown to participants in Study 1.

Participants were then asked to evaluate the level of four specific nutritional factors in the product. They reported whether they found the product to be low or high in calories, fat, salt and sugar on four separate nine-point scales (1 – Very low in [calories/nutrient] to 9 – Very high in [calories/nutrient]). On a separate page, they responded to a three-item healthiness measure asking “Based on this nutrition information, how healthy/nutritious/good do you think these potato chips are [this granola is]/will be for you?” on nine-point scales [1 – Not at all healthy/nutritious/good for me to 9 – Extremely healthy/nutritious/good for me; Cronbach’s *α* = 0.847; adapted from Hagen (34)]. Finally, they were asked how likely they would be to purchase the item (1 – Not at all likely to 9 – Extremely likely).

The study ended with participants reporting how frequently they eat either potato chips or granola depending on their experimental condition (1 – Never to 6 – Daily). In a separate part of the research session, participants also reported how frequently they read nutrition labels (1 – Never to 7 – Always) and their subjective nutrition knowledge (“How much do you know about nutrition?” from 1 – Much less than other people to 7 – Much more than other people). They also responded to a six-item health consciousness measure [Cronbach’s *α* = 0.838; Michaelidou and Hassan, (35)], and reported their subjective level of English language skill, gender and age.

### 3.2 Results

The results of a MANOVA showed that product category significantly influenced nutrition perceptions of participants (*F*(4, 245) = 9.345, *p* < 0.001, *η*_p_^2^ = 0.132, Pillai’s Trace = 0.132). The univariate results (see Figure 2 and Web Appendix A) show that participants rated the level of calories, fat and salt significantly (all *p*’s < 0.001) and sugar marginally significantly (*p* = 0.076) higher when the nutrition label was said to be associated with potato chips rather than granola. An ANOVA showed that the granola (M = 4.90, SD = 1.26) was also seen as healthier overall than the potato chips (M = 3.17, SD = 1.11; *F*(1, 248) = 132.93, *p* < 0.001, *η*_p_^2^ = 0.349) despite having identical nutritional values presented on the label. There was no significant difference in purchase likelihood across the two products, however (M_chips_ = 4.32, SD = 1.94; M_granola_ = 4.23, SD = 1.77; *F*(1, 248) = 0.155, *p* = 0.694, *η*_p_^2^ = 0.001).

**Figure 2:**
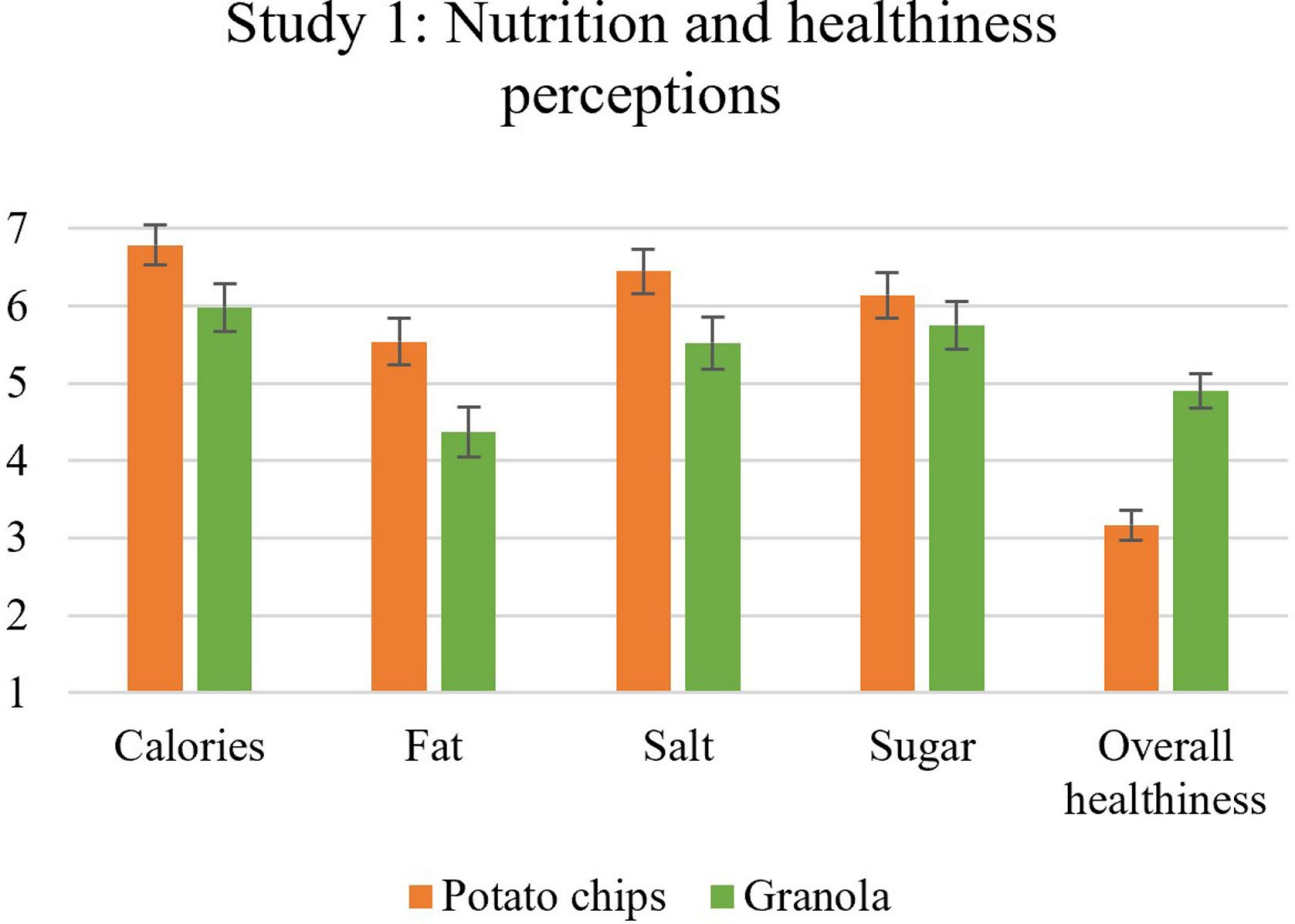
Error bars represent 95% CI. For nutrient perceptions, higher values indicate higher perceived level of the nutrient (i.e., less healthy levels). For overall healthiness, higher values indicate higher perceived healthiness.

We expected that consumers who are generally health and/or nutrition focused would rely more strongly on the specific nutrition information they see and less on the product category as a cue for healthiness. To test this, we ran additional exploratory analyses testing subjective nutrition knowledge, frequency of reading nutrition labels as well as health consciousness as potential moderators of the category healthiness effect. In separate analyses with PROCESS Model 1 [10,000 bootstraps; (36)], including the experimental condition as independent variable and overall healthiness perception as the dependent variable, we do not find a significant interaction with neither subjective nutrition knowledge (*b* = 0.081, se = 0.057, *p* = 0.157) nor nutrition label use (*b* = −0.002, se = 0.044, *p* = 0.970). However, there is a marginally significant interaction with health consciousness (*b* = 0.150, se = 0.089, *p* = 0.091). Unexpectedly, the category effect is *stronger* among those who score *higher* on health consciousness (at one SD above the mean *b* = 0.973, se = 0.098, *p* < 0.001; at one SD below the mean *b* = 0.748, se = 0.103, *p* < 0.001). However, the effect remains significant at all levels of health consciousness.

### 3.3 Discussion study 1

Study 1 provides initial evidence that consumers may evaluate the same nutrition information very differently depending on the product category. Despite seeing the same nutrition label, participants rate the product to be healthier when it is said to be from a healthier (granola) rather than less healthy (potato chips) category. For overall healthiness the pattern may simply indicate – correctly – that these four pieces of nutritional information are not the only ones that make up a product’s overall healthiness, but participants also rate the same specific value of calories, fat, salt and sugar to be higher when the information is related to potato chips than when it is associated with granola. This may indicate a category-level health halo effect, where consumers’ expectations of a broader category led them to evaluate specific nutrition information in line with those prior expectations. This can particularly be an issue in categories where products vary widely in nutritional content or where the category-level expectation does not match reality. Another potential explanation to the effect may be that consumers simply do not understand such nutritional details and thus must rely on their category perceptions to evaluate the product. We consider this in Study 2.

## 4 Study 2

Study 2 adds to the previous in two key ways. First, we use two food categories that are usually more similar to each other in nutritional content but still vary in perceived healthiness (chocolate granola bars vs. chocolate chip cookies). Second, we add a manipulation of nutritional profiles (less vs. more healthy) to test whether consumers react to the specific values on the nutritional label. If the main effect is driven by consumers not understanding nutritional information, we should find that changing the details on the nutritional label should not impact consumer evaluations, while product category would continue to do so.

### 4.1 Study design

Study 2 followed a 2 (category healthiness: healthier versus less healthy) × 2 (nutritional profile: healthier versus less healthy) between-subjects design. The procedure was similar to Study 1. Participants (203 undergraduate students^2^) were either told to imagine they came across a pack of chocolate granola bars or chocolate chip cookies and were then randomly assigned to see a nutritional label with either slightly healthier or less healthy levels of the four nutrients (see Figure 3). Two participants were excluded from the analyses based on a simple attention check asking them to select the number six as their answer, making the final sample size 201. The measures were the same as in Study 1.

**Figure 3:**
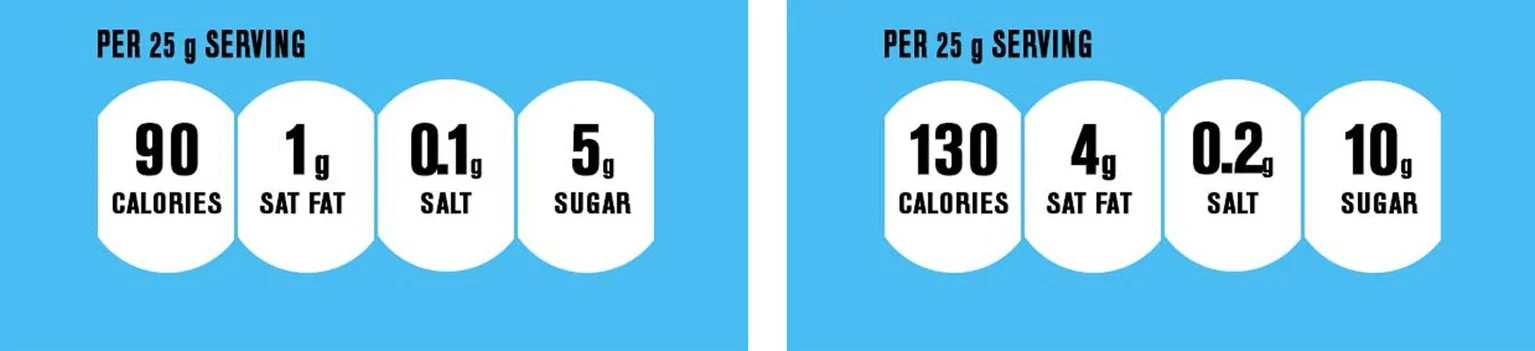
Front-of-pack labels shown to participants in Study 2. Left depicts the healthier and the right the less healthy nutritional profile.

### 4.2 Results

A MANOVA of product category and nutritional profile on the four nutrition perceptions shows a main effect of category (*F*(4, 194) = 12.964, *p* < 0.001, *η*_p_^2^ = 0.211, Pillai’s Trace = 0.211) as well as of nutritional profile (*F*(4, 194) = 3.647, *p* = 0.007, *η*_p_^2^ = 0.070, Pillai’s Trace = 0.070). No interaction between the two factors emerges (*F*(4, 194) = 0.669, *p* = 0.614, *η*_p_^2^ = 0.014, Pillai’s Trace = 0.014). The granola bar is seen as having lower levels of calories, fat, salt and sugar than the cookie (all *p*’s < 0.05) and the healthier nutritional profile is rated as healthier across each of these four factors than the less healthy profile for both products (all *p*’s < 0.05). Participants report a higher purchase likelihood for the cookies (M = 5.26, SD = 1.99) than the granola bar (M = 3.92, SD = 1.91), but their purchase likelihood is not influenced by the nutritional profile (main effect: *F*(1, 197) = 1.024, *p* = 0.313, *η*_p_^2^ = 0.005; interaction *F*(1,197) = < 0.01, *p* = 0.998, *η*_p_^2^ < 0.01). See Figure 4 for visualization and Web Appendix B for detailed statistics.

**Figure 4:**
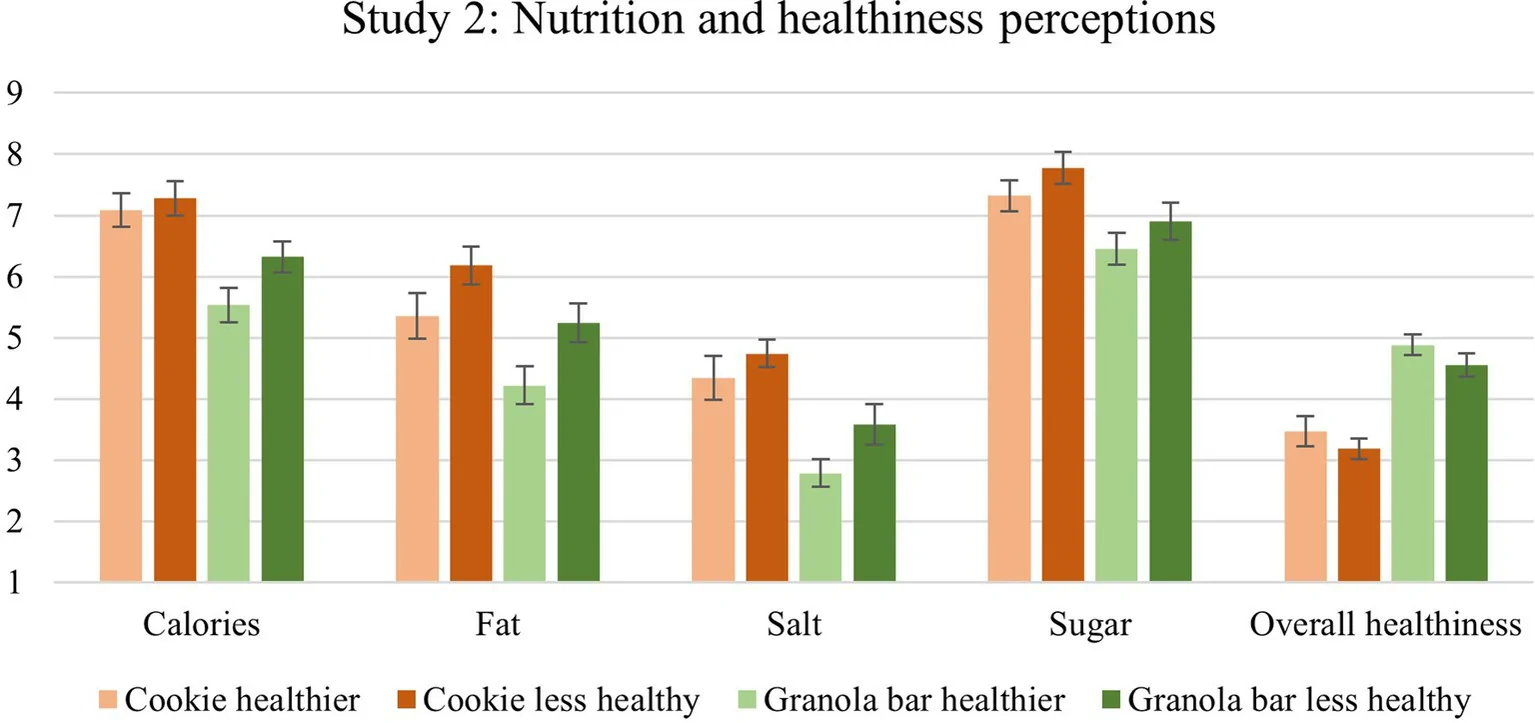
Error bars represent 95% CI. For nutrient perceptions, higher values indicate higher perceived level of the nutrient (i.e., less healthy levels). For overall healthiness, higher values indicate higher perceived healthiness.

We again explored possible interactions of product category and nutrition profile with subjective nutrition knowledge, nutrition label use and health consciousness using overall healthiness perception as a dependent variable. No three-way interaction emerges for subjective nutrition knowledge (*b* = −0.034, se = 0.057, *p* = 0.551) or nutrition label use (*b* = 0.025, se = 0.038, *p* = 0.506) with product category and nutrition profile. For health consciousness, however, significant two-way interactions of product category and nutrition profile (*b* = −0.930, se = 0.421, *p* = 0.029) as well as product category and health consciousness (*b* = 0.286, se = 0.082, *p* < 0.001) emerge, qualified by a three-way interaction of the two experimental factors and health consciousness (*b* = 0.185, se = 0.082, *p* = 0.025). The pattern (see Figure 5) shows that those *higher* on health consciousness rely *more* on the product category as a cue for healthiness evaluations, especially when the nutrition profile is relatively healthy.

**Figure 5:**
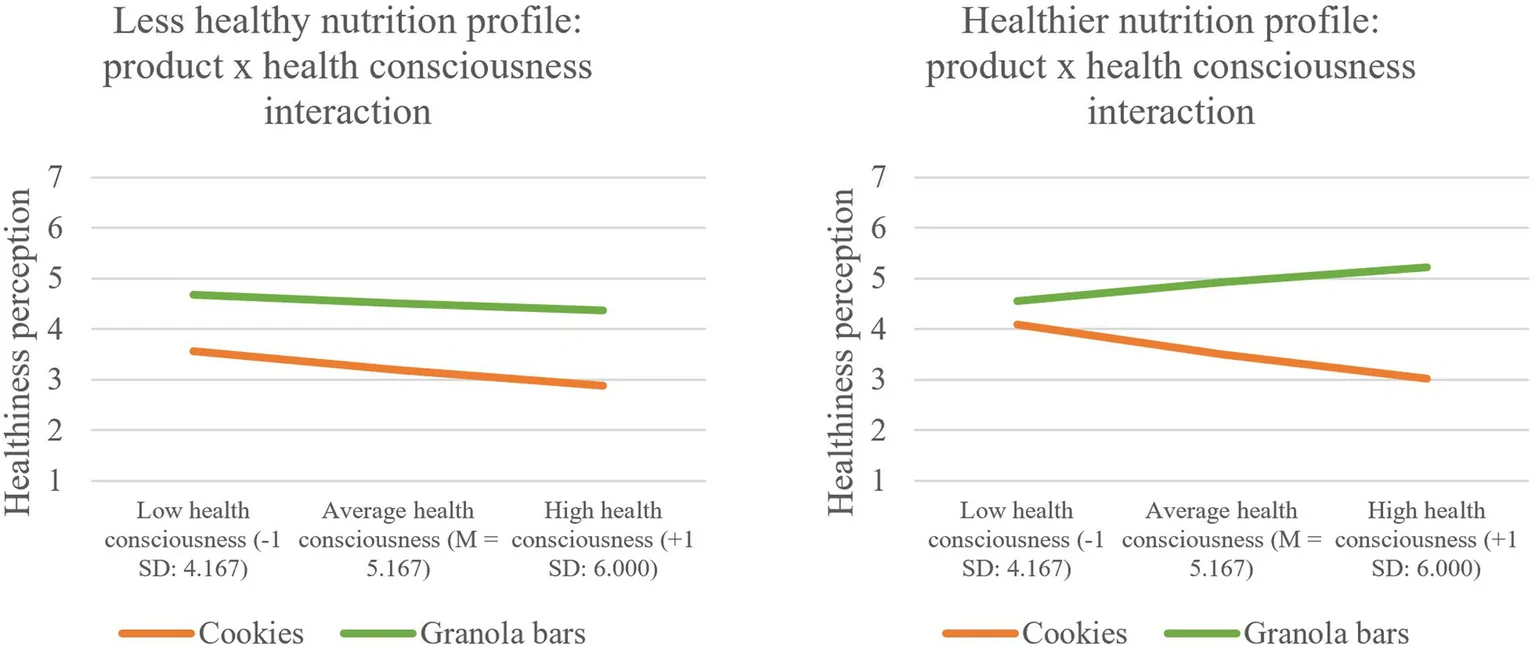
Three-way interaction of product category, nutrition profile and health consciousness in Study 2. Left depicts the category bias across levels of health consciousness for the less healthy nutrition profile, right for the healthier nutrition profile.

### 4.3 Discussion

Study 2 replicates the main effect found in Study 1 in two other product categories by showing that consumers evaluate the same nutrition information more positively when it is said to be associated with a product from a healthier category, chocolate granola bars, than when it is related to a product in a less healthy category, chocolate chip cookies. These two categories are more similar in nutritional profiles than the two used in Study 1, making this a more conservative test of the effect.

Additionally, we manipulated the details presented on the nutrition label to test whether our finding may simply be a function of participants’ difficulties in understanding nutrition information. Our findings suggest that this is not the key driver of our effect: when the values on the nutrition label were relatively more (versus less) healthy, participants also rated the product more positively. Thus, they are able to comprehend the labels and adapt their specific evaluations accordingly. However, the difference between categories remained, suggesting that the effect is more likely to be a result of consumers interpreting the nutrition information differently depending on their general expectations of the category, rather than ignoring it altogether. In Study 3, we set out to investigate this explanation more by manipulating category perceptions. Furthermore, we find additional evidence that health consciousness can influence category bias by showing that those especially high in health consciousness show a stronger bias especially when the nutrition information is relatively healthy. We return to this and test possible explanations for this pattern in Studies 4A and 4B.

## 5 Study 3

Studies 1 and 2 show that consumers evaluate identical nutrition information differently depending on the product category. Study 2 additionally shows that the effect is not driven by an ignorance towards the details on the nutrition label, but rather that the information is *interpreted* differently depending on the context. Therefore, we expect that finding ways to change consumers’ perceptions about the category should also impact their evaluation of the nutrition information.

### 5.1 Study design

Study 3 followed a largely similar procedure as the previous two studies, but instead of manipulating product category healthiness by comparing different categories, we aimed at influencing participants’ perceptions of the category’s healthiness by having them read a text about how healthy or unhealthy granola bars are (see Web Appendix C for the exact manipulation). Thus, the study followed a single-factor two-cell (category healthiness: healthy versus unhealthy) between-subjects design.

After participants (*N* = 213 undergraduate students; 43.2% female; M_age_ = 19.39) read the text about granola bars, to ensure the effectiveness of the manipulation, they were asked “How healthy do you think granola bars in general are?” (1 – Not at all healthy to 9 – Very healthy). After this, they were presented the same scenario as in the previous studies and shown the same nutrition label as in Study 1. The dependent variables and demographics were measured on the same items as in previous studies. The scales for health consciousness (Cronbach’s *α* = 0.859) and healthiness perception (Cronbach’s *α* = 0.849) were found reliable and averaged into single measures. As an attention check, participants were asked to select the number six as their response to a question. Five participants did not follow the instruction and were excluded from all analyses making the final data set consist of 208 responses (healthy condition *N* = 103, unhealthy *N* = 105).

### 5.2 Results

A one-way ANOVA showed that the manipulation of category healthiness was successful (*F*(1, 206) = 121.66, *p* < 0.001, *η*_p_^2^ = 0.371). Those who read a text about granola bars as a healthy snack rated granola bars generally as healthier (M = 6.41, SD = 1.58) than those who read a text about the unhealthy aspects of granola bars (M = 3.88, SD = 1.73).

A MANOVA of category healthiness on the four nutrition perceptions did not reach significance overall (*F*(4, 203) = 1.935, *p* = 0.106, *η*_p_^2^ = 0.037, Pillai’s Trace = 0.037). The univariate results show that when granola bars were framed as relatively unhealthy, participants rated the nutrition information less positively (i.e., higher levels of the unhealthy nutrients) but the difference only reached significance for fat (*p* = 0.028) and sugar (*p* = 0.038). See Figure 6 and Web Appendix D for details of these results.

**Figure 6:**
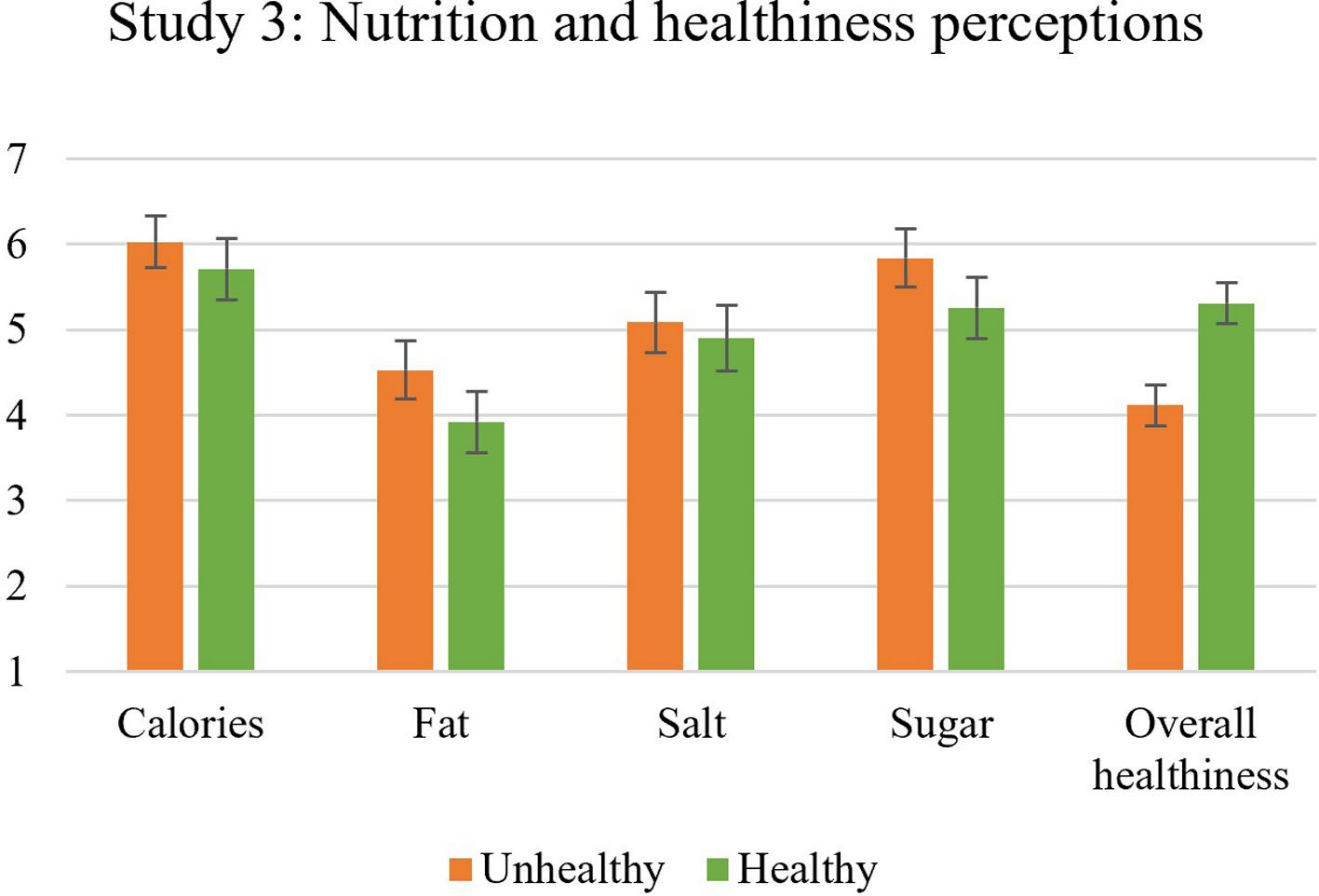
Error bars represent 95% CI. For nutrient perceptions, higher values indicate higher perceived level of the nutrient (i.e., less healthy levels). For overall healthiness, higher values indicate higher perceived healthiness.

Despite the individual nutrient evaluations being only partly influenced, a one-way ANOVA shows that the product was evaluated as healthier overall when the category was framed as healthier (M = 5.31, SD = 1.36) than less healthy (M = 4.12, SD = 1.34; *F*(1, 206) = 40.561, *p* < 0.001, *η*_p_^2^ = 0.165). Participants also reported a higher purchase likelihood for the granola bar when they had read about granola bars being healthy (M = 4.49, SD = 2.03) rather than unhealthy (M = 3.32, SD = 1.94; *F*(1, 206) = 17.828, *p* < 0.001, *η*_p_^2^ = 0.080).

We tested for the same interactions as in the previous studies, again using PROCESS macro (Model 1; 10,000 bootstraps). Neither health consciousness (b = 0.060, se = 0.103, *p* = 0.563), subjective nutrition knowledge (b = 0.035, se = 0.061, *p* = 0.571) nor nutrition label use (b = 0.042, se = 0.041, *p* = 0.303) influenced the effect of category healthiness framing on overall healthiness perception.

### 5.3 Discussion

Study 3 aimed to test more directly whether the difference in the evaluation of the nutrition labels in Studies 1 and 2 was indeed driven by the underlying expectations about the broader category’s healthiness. By framing the category of granola bars as either healthy or unhealthy, we find partial evidence for this. The framing manipulation significantly impacted the overall healthiness evaluations of the product despite the nutrition information shown to the participants being the same across conditions. However, the overall nutrition perceptions were not influenced.

The results also show that the effect on overall healthiness is not sensitive to health consciousness, subjective nutrition knowledge and nutrition label use. This indicates that regardless of consumers’ focus on health and nutrition, they seem likely to rely highly on information and knowledge regarding a product *category* when evaluating products. Product-specific information seems to play a lesser role. In the first two studies we found some initial evidence that the category bias may be stronger for the more health-conscious consumers, which would imply that those who (feel like they) focus much on health are in fact *more likely* to interpret nutrition information based on their existing perceptions rather than adapting these based on the objective information presented. In Studies 4A and 4B we turn to trying to understand this better.

## 6 Study 4A and 4B

Studies 1 and 2 show that at least in certain contexts consumers who report higher levels of health consciousness showcase a *stronger* bias based on product category. This finding is interesting as one might expect that especially those consumers who are health conscious would pay more attention to specific nutrition information on labels. A possible explanation for this finding could be in the subjective information processing of consumers. Like in other health contexts such as specific diseases (37) or risk of certain cancer (38), the judgment derived from objective information may be influenced by the consumer’s self-view. Thus, we posit that a consumer who views themself as especially knowledgeable about health may trust their subjective knowledge and perceptions more than the information provided on the front-of-pack label. If our reasoning holds, people who place higher (and maybe unjustified) confidence in their health knowledge might simply not take the label information into account when evaluating the product as they believe to already know it and rather simply use the product category as a proxy for it.

Thus, in Studies 4A and 4B we set out to test the idea that the interaction effect identified in the previous studies has to do with the respondent’s potential overconfidence in their own nutrition knowledge and measured respondents’ objective nutrition knowledge as well as their (over)confidence in this knowledge to understand how these factors may influence the category bias in interpreting front-of-package nutrition labels.

### 6.1 Study design

In both studies, the design was similar to the previous ones and participants recruited from Prolific Academic (A: N = 248, M_age_ = 36.30, 64.9% female; B: N = 248, M_age_ = 31.46, 58.5% female)^3^ were assigned to evaluate the same nutrition label but were told that it was found either on a granola bar or cookie package. We aimed for a minimum of 100 participants per experimental condition to align with the previous studies and recruited additional participants to account for possible exclusions due to missed attention checks. The label was the healthier label from Study 3. They responded to the same measures of nutrient evaluations, overall healthiness perception and purchase intention as well as background information as in previous studies. They then went through a filler task asking participants to evaluate fictional brand names on a number of characteristics.

After the filler task, participants were told they were going to be answering questions in a nutrition knowledge quiz. The questions were based on a quiz published by a UK health insurance company^4^, aligned with the UK nutrition guidelines at the time of data collection, and can be found in Web Appendix E. Across the two studies, we measured (over)confidence in two ways by changing the order of our questions. In Study 4A, participants were first asked, as a prospective estimate, “How many questions of the 10 do you expect to be able to answer correctly?” (M = 5.82, SD = 1.90) and then given the quiz. In Study 4B, participants first took the quiz and then were asked, as a retrospective estimate, “How many questions do you think you answered correctly in the quiz you just took?” (M = 4.51, SD = 1.70). In both studies, we used the number of correct answers as a measure of objective nutrition knowledge, and calculated (over)confidence as the difference between participants’ expected score and their actual score (where positive values indicate overconfidence and negative values underconfidence in their nutrition knowledge). The participants were then shown the correct answers and thanked for their participation.

### 6.2 Results

Both studies again replicate the effect of product type. In Study 4A both the MANOVA on nutrient perceptions (*F*(4, 243) = 6.031, *p* < 0.001, *η*_p_^2^ = 0.090, Pillai’s Trace = 0.090) and ANOVA on overall healthiness perception (*F*(1, 246) = 57.784, *p* < 0.001, *η*_p_^2^ = 0.190) show that the granola bar is perceived as containing lower levels of the nutrients presented on the label and being more healthy than the cookie despite the nutrition information provided being the same. Also in Study 4B both the MANOVA on nutrient perceptions (*F*(4, 243) = 7.561, *p* < 0.001, *η*_p_^2^ = 0.111, Pillai’s Trace = 0.111) and the ANOVA on overall healthfulness (*F*(1, 246) = 61.352, *p* < 0.001, *η*_p_^2^ = 0.200) show strong and significant differences across the two products. See Figure 7 for visualization of the effects and Web Appendix F for details on the univariate results.

**Figure 7:**
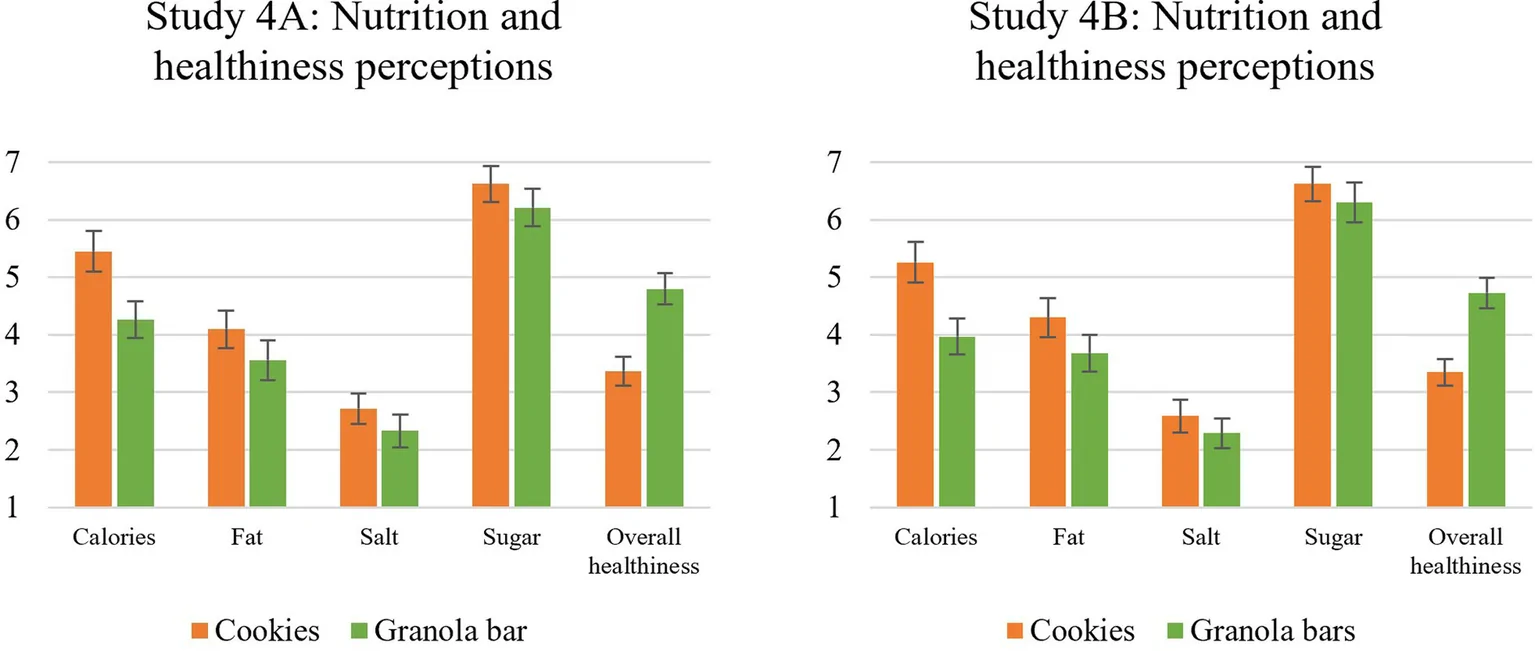
**(A)** for Study 4A; **(B)** for Study 4B. Error bars represent 95% CI. For nutrient perceptions, higher values indicate higher perceived level of the nutrient (i.e., less healthy levels). For overall healthiness, higher values indicate higher perceived healthiness.

However, we do not find the interaction of knowledge confidence and product type on healthfulness perceptions as we expected (Study 4A: *b* = 0.043, se = 0.042, *p* = 0.309; Study 4B: b = 0.038, se = 0.044, *p* = 0.400). Interestingly, there is no interaction of product type and *objective* knowledge either (Study 4A: b = −0.015, se = 0.078, *p* = 0.843; Study 4B: b = −0.008, se = 0.073, *p* = 0.912).

### 6.3 Discussion

Studies 4A and 4B again replicate the category bias effect by showing that consumers evaluate the same nutrition information to be indicative of a more healthful product when it is said to be from a healthier (granola bars) versus less healthy (cookies) category. This speaks to the robustness of the effect and the strong role that consumers’ category perceptions play when evaluating product healthiness.

Perhaps concerningly, we do not find that consumers’ objective nutrition knowledge helps overcome this halo either. While it may be that overall healthiness perceptions are based on evaluations of more factors than the four pieces of nutritional information presented to the participants, this cannot account for the lack of effect of nutrition knowledge, as the category bias also appears for individual nutrient evaluations. Consumers who were able to answer nutrient-specific questions in the quiz would be expected to evaluate for example calories per portion more objectively. Yet even those with higher quiz scores still showed evaluations shaped by the product type on which the label appeared. In other words, stronger nutrition knowledge may be limited in helping overcome the category bias.

Furthermore, contrary to our expectations, we do not find any consistent evidence that the category bias would be strengthened by a higher level of (over)confidence in one’s nutrition knowledge. Given the indications that the bias may be stronger for those higher in health consciousness from earlier studies, we expected that this may be caused by the more health-conscious consumers relying more strongly on their own internal knowledge of product categories over nutrition label information. However, despite using two different measures of knowledge confidence, we cannot conclude that this would explain the interaction with health consciousness. Overall, the results show that the category bias is strong and goes above many other factors in influencing product and nutrition evaluations.

## 7 General discussion

Front-of-package labels are increasingly popular and often seen as a key tool to promoting healthier choices among consumers. However, previous research has revealed that their impact on consumer choice may be more limited than policy makers hope for (1, 39). In the present work, we propose that one reason for their limited effectiveness may be a bias in consumers’ interpretation of objective nutrition information. Across five studies we show that nutrition information is interpreted in the context of the product it is placed on such that the evaluation of the nutritional levels is in line with one’s expectations regarding a product category’s healthiness. In all our studies, the exact same nutrition label results in higher perceptions of overall healthiness and lower perceptions of the presence of undesirable nutrients^5^ when it is said to be placed on a product from a relatively healthy (e.g., granola bar) than from a relatively unhealthy category (e.g., cookies).

We further show that the effect is not driven by a lack of understanding of the nutrition label (Study 2): consumer perceptions of healthiness are influenced by the details of the nutrition label (i.e., healthier versus less healthy levels of nutrients presented), but the category bias remains. Rather, our findings are in line with research on confirmation bias, which suggests that consumers tend to interpret information in line with their own expectations (e.g., 24, 25). In our context, participants interpret nutrition information in line with their prior expectations regarding a product category. In fact, adjusting their prior expectations using a magazine article also changes their interpretation of the nutrition information in terms of overall healthiness as well as some nutrients (Study 3). This is similar to the findings of Gonzales et al. (26), who also showed that by educating consumers about the nutritional comparison between products typically seen as healthy or unhealthy, such a bias can be mitigated. However, the question remains how persistent such effects are over time.

Finally, we find initial evidence that the category bias effect is stronger among those who report higher subjective health consciousness (Study 1, Study 2). However, at least within our experimental context, this does not seem to be related neither to a higher level of objective nor subjective nutrition knowledge, nor overconfidence regarding one’s knowledge about nutrition (Study 4A and 4B). Crucially, even those with high levels of objective nutrition knowledge still show the same category bias in their interpretations of the nutrition label. As these findings are not conclusive, and not fully consistent across the studies, more work into understanding the role of individual differences in the use and interpretation of nutrition knowledge is needed. However, the notion that the category bias seems persistent across all levels of nutrition knowledge in our study context is highly problematic as increasing the nutritional knowledge of consumers has often been offered as a key way to promote healthier decisions (40).

### 7.1 Theoretical contributions

Our work adds to previous research on health halos (e.g., 2, 26) by showing that even when identical nutrition information is presented, this is interpreted differently when consumers believe it to be associated with a product from a relatively healthy (vs. unhealthy) category. This also provides insight into the role of confirmation bias in the context of nutrition labeling.

Overall, our findings highlight the importance of carefully considering the product category that is used when studying the effects of nutrition labels: findings are likely to be highly dependent on consumers’ expectations regarding the healthiness of the category. Additionally, even though our findings are preliminary, we show the importance of considering individual differences in this context and add to the limited research on nutrition knowledge and (over) confidence (e.g., 39). Our findings suggest there may be a negative consequence of increasing health consciousness, which could make consumers rely even more on their own intuition over objective nutrition information. This warrants more research in the future.

### 7.2 Practical contributions

Policy makers and marketers of healthier products especially in categories perceived as less healthy should focus on educating consumers about category healthiness so that these perceptions do not lead to incorrect assumptions about specific products. Our results suggest that without such efforts, even objective nutrition labels may be interpreted in line with category stereotypes rather than on their own merits. Potentially, it may be helpful to remind consumers that a product may *not* be representative of the entire category or in other ways promote more conscious processing of nutrition information. However, this remains a difficult task given the limited time consumers have when making their purchase decisions in the supermarket. Policy makers could therefore explore interventions at the point of purchase, such as simplified FOP labels or warnings when a product deviates strongly from category norms, to make the “mismatch” salient without requiring extensive comparisons. Recent work (41) provides initial evidence that more interpretive labels such as the Nutri-Score and warning labels can help consumers adjust their healthiness perceptions of products. Especially warning labels were found to hold good promise as being effective in breaking down health halos. This is especially relevant as highly unhealthy product variants in relatively healthy categories may be especially harmful and misleading to consumers based on our findings.

### 7.3 Future research and limitations

Our research was conducted in highly controlled experimental conditions, but we propose that this makes our findings potentially more a conservative interpretation. Participants were only shown nutrition information, and were not distracted by other information on packaging, other products, people or real time pressure, which would suggest that their ability to focus on the presented nutrition information was especially strong in comparison to real-life store environments. In the store, with the numerous factors competing for attention, reliance on intuition and previous experience may be further exacerbated. On the other hand, highly motivated consumers do have better opportunities to make direct comparisons between products, which can reduce the category bias. However, the proportion of consumers who are able and willing to make this effort is low and we expect the bias we identify in this paper to remain a major issue for most consumers in the retail environment as well. Notably, we have not tested the effect in a real shopping situation, limiting the ecological validity of our studies, and we therefore propose this as an avenue for future research. This would further allow testing the robustness of our findings in a more representative sample beyond undergraduate students and panel participants, and for actual purchase behavior rather than intentions only. Relatedly, our purchase measure captured stated purchase likelihood on a single item in a hypothetical scenario. Therefore, it cannot be interpreted as evidence of actual purchasing behavior. Future research should address this limitation with consequential behavioral measures.

Our research provides first insights into the possible moderating role of health consciousness. Interestingly, we do not find similar patterns with subjective or objective nutrition knowledge nor (over)confidence. Rather than treating these as inconclusive null findings, one can see them as support for the idea that knowledge alone may not be sufficient to counteract intuitive, category-based judgments. These findings are preliminary and warrant further research to gain better insight into the role of these individual differences in mitigating or exacerbating biases relating to the interpretation of nutrition information. However, we show that a stronger health consciousness may even prohibit consumers from taking nutrition information into account. Future research should test the robustness of this finding, which was not fully consistent across our studies, and investigate what may be causing this effect. For instance, could it be that highly health-conscious consumers mistrust nutrition labels, or that knowledge and confidence only matter under certain situational conditions (e.g., time pressure, label format)? Importantly, future work should go beyond detecting *no effect* and directly test whether knowledge truly plays no role in moderating the category bias.

Finally, while our findings propose that the category bias can be to some extent mitigated by changing consumers perceptions regarding the healthiness of the category, this is not always practical. Firstly, consumers may hold strong beliefs about a category, making adjusting these perceptions difficult. Secondly, products within a category can vary strongly in their nutritional value. This means that future research should look at other ways to mitigate the category bias, not only through adjusting category-level perceptions, but also through prompting consumers to evaluate products as individual items and not only in relation to their product category.

In conclusion, our findings robustly show that consumers interpret objective nutrition information differently depending on the category it is associated with. While we also find that consumers are able to adjust their perceptions based on the actual values on the label, the category bias is persistent across varying nutritional levels of products as well as nutrition knowledge of consumers. Notably, within the conditions of our research, we do not find evidence that nutrition knowledge or confidence attenuates this bias, highlighting that even well-informed consumers may remain susceptible to the category bias effect. This work provides a new perspective towards understanding the mixed findings regarding the effectiveness of front-of-package nutrition labels as drivers of healthier purchase decisions.

## Data Availability

The datasets presented in this study can be found in online repositories. The names of the repository/repositories and accession number(s) can be found at: https://researchbox.org/4685.

## Footnotes

1
https://researchbox.org/4685

2 Demographic measures were not collected due to error in the survey software, but participants are from the same subject pool as in Study 1. However, participants who already participated in Study 1 were not able to take part in Study 2. The varying number of participants per experimental condition in comparison to other studies in this paper is due to participant availability within the subject pool.

3 The studies included two attention checks, one after the first block asking participants to select the number six as their response and another among the nutrition quiz asking participants to select the highest number of the options as their answer. In Study 4A, three of the 251 participants failed the latter attention check and are excluded from all analyses, leading to the final N of 248. In Study 4B, one of the 251 participants failed the first and two participants the second attention check, leading to the final N of 248.

4 The approach to measuring objective nutrition knowledge through a quiz was inspired by Andrews, Netemeyer and Burton’s (42) work on consumer interpretation of nutrition claim information. The quiz was chosen based on a pre-test of two similar nutrition quizzes. We chose the option where the average score was closer to 50% to allow for sufficient variation in the scores to study effects of nutrition knowledge.

5 Study 3 supports this pattern only in part, while directionally consistent and significant for two of the four nutrients individually, the effect on overall nutrition perceptions did not reach significance. This weaker result in comparison to the other studies may be due to the more subtle manipulation, where the category remained that same and healthiness was framed by means of a text. Nevertheless, this highlights that our effect may not be universal to all nutrients in all situations and calls for more research into the boundary conditions of the category effect on individual nutrients.
