# Supplementary material for: Category bias and front-of-package labelling
Source: Front Public Health. 2026 Oct 27;14:1963968. doi: 10.3389/fpubh.2026.1963968 (PMC13750253; doi:10.3389/fpubh.2026.1963968)
Supplement: Supplementary file 1 [file Supplementary_file_1.docx]

**Category bias and front-of-package labelling**

**Supplementary materials**

**Table of** **Contents**

[Web Appendix A: Key statistics Study 1	ii](#_Toc210738895)

[Web Appendix B: Key statistics Study 2	iii](#_Toc210738896)

[Web Appendix C: Healthiness manipulation Study 3	iv](#_Toc210738897)

[Web Appendix D: Key statistics Study 3	vi](#_Toc210738898)

[Web Appendix E: Knowledge quizzes Studies 4A and 4B	vii](#_Toc210738899)

[Web Appendix F: Key statistics Studies 4A and 4B	ix](#_Toc210738900)

# Web Appendix A: Key statistics Study 1

**Table A1.** Key statistics for all dependent variables in Study 1.

|  | Potato chips | | Granola | | Univariate tests | | |
| --- | --- | --- | --- | --- | --- | --- | --- |
|  | Mean | SD | Mean | SD | Test statistics | p-value | Effect size (η­_p_^2^) |
| Calories | 6.79 | 1.46 | 5.98 | 1.79 | F(1, 248) = 15.483 | < .001 | .059 |
| Fat | 5.54 | 1.73 | 4.37 | 1.81 | F(1, 248) = 27.586 | < .001 | .100 |
| Salt | 6.45 | 1.63 | 5.52 | 1.91 | F(1, 248) = 17.395 | < .001 | .066 |
| Sugar | 6.14 | 1.65 | 5.75 | 1.75 | F(1, 248) = 3.177 | .076 | .013 |
| Healthiness | 3.17 | 1.11 | 4.90 | 1.26 | F(1, 248) = 132.93 | < .001 | .349 |
| Purchase likelihood | 4.32 | 1.94 | 4.23 | 1.77 | F(1, 248) = .155 | .694 | .001 |

# Web Appendix B: Key statistics Study 2

**Table B1.** Means and SDs for all dependent variables in Study 2.

|  | Chocolate chip cookies | | | | Chocolate granola bars | | | |
| --- | --- | --- | --- | --- | --- | --- | --- | --- |
|  | Healthier | | Less healthy | | Healthier | | Less healthy | |
|  | Mean | SD | Mean | SD | Mean | SD | Mean | SD |
| Calories | 7.08 | 1.56 | 7.28 | 1.16 | 5.53 | 1.59 | 6.32 | 1.44 |
| Fat | 5.36 | 2.12 | 6.18 | 1.47 | 4.22 | 1.75 | 5.24 | 1.81 |
| Salt | 4.34 | 2.06 | 4.74 | 1.82 | 2.78 | 1.27 | 3.58 | 1.86 |
| Sugar | 7.32 | 1.44 | 7.78 | 1.18 | 6.45 | 1.47 | 6.90 | 1.69 |
| Healthiness | 3.47 | 1.42 | 3.18 | 1.06 | 4.88 | 0.97 | 4.55 | 1.07 |
| Purchase likelihood | 5.40 | 1.99 | 5.12 | 2.01 | 4.06 | 2.12 | 3.78 | 1.68 |

**Table B2.** Key statistics for all dependent variables in Study 2.

|  | Main effect of category | | | Main effect of nutrition profile | | | Interaction | | |
| --- | --- | --- | --- | --- | --- | --- | --- | --- | --- |
|  |  |  |  |  |  |  |  |  |  |
|  | Test statistics | p-value | Effect size (η­_p_^2^) | Test statistics | p-value | Effect size (η­_p_^2^) | Test statistics | p-value | Effect size (η­_p_^2^) |
| Calories | F(1, 197) = 37.735 | < .001 | .161 | F(1, 197) = 5.875 | .016 | .029 | F(1, 197) = 2.088 | .150 | .010 |
| Fat | F(1, 197) = 16.834 | < .001 | .079 | F(1, 197) = 13.180 | < .001 | .063 | F(1, 197) = .162 | .688 | .001 |
| Salt | F(1, 197) = 29.453 | < .001 | .130 | F(1, 197) = 5.710 | .018 | .028 | F(1, 197) = .625 | .430 | .003 |
| Sugar | F(1, 197) = 18.085 | < .001 | .084 | F(1, 197) = 4.885 | .028 | .024 | F(1, 197) = .001 | .979 | < .001 |
| Healthiness | F(1, 197) = 74.527 | < .001 | .247 | F(1, 197) = 3.729 | .055 | .019 | F(1, 197) = .012 | .912 | <.001 |
| Purchase likelihood | F(1, 197) = 23.572 | < .001 | .107 | F(1, 197) = 1.024 | .313 | .005 | F(1, 197) < .01 | .998 | < .001 |

# Web Appendix C: Healthiness manipulation Study 3

**Healthy condition**

**Are granola bars healthy?**

Many people consider granola bars a convenient and healthy snack and enjoy their flavor and versatility. Granola bars are not only convenient, budget-friendly, and portable but also often a healthy alternative to other snacks. Not to mention, granola bars contain healthy ingredients like oats, nuts, seeds, and dried fruit, which can make a beneficial addition to any diet.

"Muesli bars can be a fantastic snack option. They are relatively inexpensive, convenient and can be a good source of vitamins, minerals and fibre," dietitian Kaitlyn Bruschi told The Huffington Post Australia.

In particular, oats – the key ingredient of granola bars - are a great source of beta-glucan, a type of fiber that can help decrease levels of total and LDL (bad) cholesterol, two risk factors for heart disease. Meanwhile, nuts, seeds, and dried fruit have been shown to benefit blood sugar control and heart health. Granola bars typically contain a good amount of protein and fiber with little added sugar and fewer calories.

**Unhealthy condition**

**Are granola bars healthy?**

Many people consider granola bars a convenient but unhealthy snack and enjoy their flavor and versatility. Granola bars are not only convenient, budget-friendly, and portable but on the other hand can also be an unhealthy alternative to other snacks. Not to mention, granola bars contain unhealthy ingredients like sugar, saturated fat and artificial sweeteners, which can make a harmful addition to any diet.

"Muesli bars can be a bad snack option. They are relatively inexpensive, convenient, but can be a poor source of vitamins, minerals and fibre," dietitian Kaitlyn Bruschi told The Huffington Post Australia.

In particular, artificial sweeteners – common ingredients in granola bars – may interfere with blood sugar control and negatively influence your beneficial gut bacteria and cause digestive issues. Meanwhile, high consumption of processed and sugary foods can increase your risk of metabolic syndrome, which influences your blood sugar control and heart health. Granola bars typically contain a limited amount of protein and fiber with added sugar and more calories.

# Web Appendix D: Key statistics Study 3

**Table D1.** Key statistics for all dependent variables in Study 3.

|  | Unhealthy framing | | Healthy framing | | Univariate tests | | |
| --- | --- | --- | --- | --- | --- | --- | --- |
|  | Mean | SD | Mean | SD | Test statistics | p-value | Effect size (η­_p_^2^) |
| Calories | 6.03 | 1.70 | 5.71 | 2.06 | F(1, 206) = 1.501 | .222 | .007 |
| Fat | 4.53 | 1.96 | 3.92 | 2.01 | F(1, 206) = 4.927 | .028 | .023 |
| Salt | 5.09 | 2.00 | 4.90 | 2.17 | F(1, 206) = .400 | .528 | .002 |
| Sugar | 5.84 | 1.93 | 5.26 | 2.05 | F(1, 206) = 4.343 | .038 | .021 |
| Healthiness | 4.12 | 1.34 | 5.31 | 1.36 | F(1, 206) = 40.561 | < .001 | .165 |
| Purchase likelihood | 3.32 | 1.94 | 4.49 | 2.03 | F(1, 206) = 17.828 | < .001 | .080 |

# Web Appendix E: Knowledge quizzes Studies 4A and 4B

**Knowledge quiz Studies 4A and 4B**
*Correct answers bolded here, but not during the actual study, where they were revealed to participants at the end of the study*

1. How much calcium does your body need each day?

- 400 mg
- 100 mg
- **700 mg**
- 100 g
- Don't know

2. How many 200ml glasses of water should an adult drink each day?

- 12 for men, or 10 for women
- **10 for men, or 8 for women**
- 8 for men, or 6 for women
- 6 for men, or 4 for women
- Don't know

3. Which of these foods doesn’t count towards your five-a-day?

- Sweet potatoes
- Parsnips
- **Potatoes**
- Orange juice
- Don't know

4. How many portions of fish should you eat each week?

- 8
- 6
- 4
- **2**
- Don't know

5. For a food to be considered ‘low-sugar’, what’s the maximum amount of sugar that it can contain?

- **No more than 5g per 100g for food and 2.5g per 100ml for drink**
- No more than 7.5g per 100g for food and 5g per 100ml for drink
- No more than 10g per 100g for food and 7.5g per 100ml for drink
- No more than 12.5g per 100g for food and 10g per 100ml for drink
- Don't know

6. How many grams of salt is the recommended daily allowance for adults in the UK?

- 5 g
- **6 g**
- 7 g
- 8 g
- Don't know

7. One gram of protein provides four calories of energy. One gram of carbohydrate provides 3.75 calories. But how many calories does one gram of fat provide?

- 2
- 3
- 8
- **9**
- Don't know

8. Where does most of your vitamin D come from?

- Eggs
- Fortified breakfast cereal
- **Sunlight**
- Carbohydrates
- Don't know

9. Which of these take-away coffees has the most calories?

- **Medium skimmed mocha**
- Medium skimmed cappuccino
- Medium skimmed latte
- Medium skimmed vanilla latte
- Don't know

10. What’s the recommended daily intake of fibre for an adult?

- 17 g
- **18 g**
- 19 g
- 20 g
- Don't know

# Web Appendix F: Key statistics Studies 4A and 4B

**Table F1.** Key statistics for all dependent variables in Study 4A.

|  | Cookies | | Granola bar | | Univariate tests | | |
| --- | --- | --- | --- | --- | --- | --- | --- |
|  | Mean | SD | Mean | SD | Test statistics | p-value | Effect size (η­_p_^2^) |
| Calories | 5.45 | 1.99 | 4.26 | 1.83 | F(1, 246) = 23.841 | < .001 | .088 |
| Fat | 4.10 | 1.85 | 3.56 | 1.99 | F(1, 246) = 4.953 | .027 | .020 |
| Salt | 2.72 | 1.51 | 2.33 | 1.63 | F(1, 246) = 3.934 | .048 | .016 |
| Sugar | 6.62 | 1.76 | 6.21 | 1.85 | F(1, 246) = 3.105 | .079 | .012 |
| Healthiness | 3.37 | 1.42 | 4.80 | 1.54 | F(1, 246) = 57.784 | < .001 | .190 |
| Purchase likelihood | 5.23 | 2.02 | 4.37 | 2.30 | F(1, 246) = 9.788 | .002 | .038 |

**Table F2.** Key statistics for all dependent variables in Study 4B.

|  | Cookies | | Granola bar | | Univariate tests | | |
| --- | --- | --- | --- | --- | --- | --- | --- |
|  | Mean | SD | Mean | SD | Test statistics | p-value | Effect size (η­_p_^2^) |
| Calories | 5.26 | 1.99 | 3.97 | 1.78 | F(1, 246) = 29.260 | < .001 | .106 |
| Fat | 4.30 | 1.91 | 3.68 | 1.81 | F(1, 246) = 6.912 | .009 | .027 |
| Salt | 2.59 | 1.61 | 2.29 | 1.49 | F(1, 246) = 2.317 | .129 | .009 |
| Sugar | 6.62 | 1.74 | 6.30 | 2.00 | F(1, 246) = 1.854 | .175 | .007 |
| Healthiness | 3.35 | 1.29 | 4.73 | 1.48 | F(1, 246) = 61.352 | < .001 | .200 |
| Purchase likelihood | 5.21 | 1.99 | 4.84 | 2.13 | F(1, 246) = 2.012 | .157 | .008 |
